# Role of Lifestyle and Nutrition in Menstrual Cycle Regularity: Associations with Body Composition and Dietary Habits

**DOI:** 10.3390/jcm15072613

**Published:** 2026-03-29

**Authors:** Angela Andreoli, Eugenia Costantini, Qeta Megan, Artida Pashaj, Ersilia Buonomo, Emilio Piccione, Maria De Bonis, Francesco Giuseppe Martire

**Affiliations:** 1 Human Physiology and Nutrition Unit, Department of System Medicine, University of Rome “Tor Vergata”, 00133 Rome, Italy; angela.andreoli@uniroma2.it; 2 Faculty of Medicine, Catholic University of Our Lady of Good Counsel, 1026 Tirana, Albania; meganqeta@gmail.com (Q.M.); a_avdia@hotmail.com (A.P.); ersiliabuonomo@gmail.com (E.B.); 3 Obstetrics and Gynecological Clinic, Department of Molecular and Developmental Medicine, University of Siena, 53100 Siena, Italy; eugenia.costantini22@gmail.com (E.C.); mariadebonis@gmail.com (M.D.B.); francescogmartire@libero.it (F.G.M.); 4 Department of Biomedicine and Prevention, University of Rome “Tor Vergata”, 00133 Rome, Italy; 5 Gynecology and Obstetrics, Department of Surgical Sciences, University of Rome “Tor Vergata”, 00133 Rome, Italy

**Keywords:** menstrual irregularity, body composition, lifestyle factors

## Abstract

**Background**: Nutritional status and lifestyle factors are increasingly recognized as relevant modulators of women’s reproductive health. However, data remain limited on the relationship between body composition, dietary habits, and menstrual cycle characteristics in apparently healthy young women. This study aimed to assess nutritional status, body composition, and lifestyle behaviors in young women and to explore their associations with menstrual cycle regularity. **Methods**: This cross-sectional study included 49 apparently healthy women aged 19–30 years. Anthropometric measurements and body composition were assessed using bioelectrical impedance analysis. Dietary habits were evaluated through a simplified food frequency questionnaire, and adherence to the Mediterranean diet was assessed using the PREDIMED score. Physical activity was estimated using MET values based on the Compendium of Physical Activities. Menstrual cycle characteristics were collected via questionnaire. Group comparisons were performed between women with regular and irregular menstrual cycles. **Results**: The sample was predominantly normal-weight (mean BMI 22.36 ± 4.26 kg/m^2^). Anthropometric and bioelectrical impedance parameters did not differ significantly between women with regular and irregular cycles. Women with irregular cycles showed higher resistance and extracellular water and lower phase angle and body cell mass, although differences were not statistically significant. A significant association was found for meat consumption, which was lower in women with irregular cycles (*p* = 0.007). No associations were observed for other dietary variables, physical activity, or meal frequency. **Conclusions**: Menstrual regularity in young women was not associated with major anthropometric differences but may be linked to subtle aspects of nutritional status and dietary habits. Lower meat consumption emerged as a potential dietary factor associated with menstrual irregularity. Although associations were modest, these findings support the relevance of nutritional and lifestyle factors in menstrual health. Larger longitudinal studies are needed to clarify these relationships.

## 1. Introduction

Nutrition plays a fundamental role in human health and represents a key determinant in the prevention of chronic diseases [1]. In young adulthood, dietary habits and lifestyle behaviors contribute significantly to long-term metabolic health, body composition, and functional status [2,3]. In women, nutritional status is closely linked to reproductive health. Recent evidence also highlights the role of nutrition in gynecological disorders such as endometriosis, where dietary factors may influence inflammation, pain, and disease progression [4]. Both undernutrition and excess adiposity have been associated with hormonal imbalances, irregular cycles, impaired ovulatory function, and reduced fertility. Hormone-dependent gynecological conditions such as endometriosis and adenomyosis are increasingly recognized as multifactorial disorders in which lifestyle and metabolic factors may play a contributory role [5]. Alterations in body fat distribution, hydration status, and cellular integrity may influence ovarian physiology and menstrual cycle characteristics. Obesity-related insulin resistance and changes in inflammatory and endocrine pathways are recognized contributors to reproductive dysfunction, including anovulation and polycystic ovary syndrome [6,7]. Lifestyle and dietary patterns may influence reproductive physiology through several pathophysiological pathways. Metabolic disturbances such as insulin resistance and chronic low-grade inflammation can alter ovarian steroidogenesis and disrupt hypothalamic–pituitary–ovarian axis regulation [6,7]. These mechanisms are particularly relevant in polycystic ovary syndrome (PCOS), a common endocrine disorder associated with irregular cycles, infertility, and increased long-term cardiometabolic risk including type 2 diabetes, dyslipidemia, and cardiovascular disease [6,7]. Beyond body weight alone, body composition assessment provides more detailed insights into nutritional and functional status. Parameters such as fat mass, fat-free mass, total body water, body cell mass, and phase angle are increasingly used as markers of metabolic health, cellular integrity, and nutritional adequacy. Bioelectrical impedance analysis represents a practical and non-invasive method to evaluate these components in population-based studies [8,9]. Adherence to healthy dietary models, such as the Mediterranean diet, and regular physical activity have been associated with improved metabolic profiles and reduced risk of menstrual disorders [10,11,12]. Nutritional factors have also been implicated in estrogen-dependent conditions such as uterine fibroids, supporting a broader link between diet and gynecological health [13,14]. Despite growing evidence, data on the relationship between lifestyle, body composition, and menstrual cycle characteristics in young female populations remain limited. However, most available studies have focused on clinical populations or specific disorders such as PCOS, while limited data are available on apparently healthy young women from general populations. Therefore, the present study aimed to assess nutritional status and lifestyle behaviors in a population of young women and to investigate the associations between dietary habits, physical activity, body composition parameters, and menstrual cycle characteristics.

## 2. Material and Methods

### 2.1. Study Population

This cross-sectional study included 49 young women aged 19–30 years recruited at the Università Cattolica Nostra Signora del Buon Consiglio (UNIZKM).

Participants were apparently healthy young women recruited on a voluntary basis. No participants reported diagnosed chronic diseases affecting nutritional or reproductive status. However, detailed clinical screening for specific endocrine conditions such as thyroid disorders, or hyperprolactinemia was not performed within the scope of this study. The study protocol was approved by the local Ethics Committee (Protocol Number 200), and all participants provided written informed consent prior to enrollment, in accordance with the Declaration of Helsinki.

### 2.2. Anthropometric Measurements

Body weight and height were measured using standard procedures. Body Mass Index (BMI) was calculated as weight divided by height squared (kg/m^2^). Body composition was assessed using bioelectrical impedance analysis (BIA) with a BIA 101 analyzer (RJL Systems, Florence, Italy). Measurements were performed in the supine position using a tetrapolar electrode configuration at 50 kHz. Resistance (R), reactance (Xc), and phase angle (PA) were recorded.

Total body water (TBW) was estimated using the equation by Lukaski et al. [15], extracellular water (ECW) using the equation by Segal et al. [16], and intracellular water (ICW) was calculated as TBW-ECW. Fat mass, fat-free mass, body cell mass, and related indices (FFMI, BCMI) were derived accordingly.

### 2.3. Lifestyle and Dietary Assessment

Lifestyle information was collected using a structured self-administered questionnaire developed for this study to capture demographic, dietary, and physical activity habits. Dietary habits were assessed using a simplified food frequency questionnaire designed to record weekly consumption frequencies of major food groups and daily meal distribution. Adherence to the Mediterranean diet was evaluated using the PREDIMED score [17]. Physical activity was quantified using weekly hours of activity and corresponding MET values based on the Compendium of Physical Activities [18,19].

### 2.4. Menstrual Cycle Assessment

Information on menstrual cycle characteristics, including cycle length, perceived regularity, dysmenorrhea, menstrual pain, intermenstrual bleeding, and secondary amenorrhea, was collected through a structured self-report questionnaire [20]. According to established criteria for normal menstrual cycles in reproductive-age women (typically 24–38 days with limited variability) [21], menstrual cycles were classified as regular when participants reported a consistent and predictable cycle pattern within this range. Cycles were classified as irregular when participants reported variability in cycle length, cycles outside the normal range, or the presence of amenorrhea, based on self-reported menstrual history. Information on hormonal therapy or hormonal contraceptive use was not systematically collected and was therefore not included in the analysis. For consistency, the terms “regular menstrual cycles” and “irregular menstrual cycles” are used throughout the manuscript to define the study groups.

### 2.5. Statistical Analysis

Statistical analyses were performed using SPSS (version 25.0; IBM Corp., Armonk, NY, USA). Data distribution was assessed using the Kolmogorov–Smirnov test. Continuous variables were expressed as mean ± SD or median (min–max), as appropriate, while categorical variables were reported as frequencies and percentages. Associations were evaluated using Pearson’s correlation, group comparisons using Student’s *t*-test or one-way ANOVA, and categorical variables using the chi-square or Fisher’s exact test, as appropriate. A *p* < 0.05 was considered statistically significant.

## 3. Results

The analysis included 49 young women aged between 19 and 30 years. The mean age of the sample was 21.7 ± 1.8 years. Participants were on average 164.9 ± 7.3 cm tall and weighed 60.9 ± 12.4 kg, with a mean body mass index (BMI) of 22.4 ± 4.3 kg/m^2^. According to WHO criteria, the sample was predominantly within the normal-weight range, indicating an apparently healthy and relatively homogeneous population [22].

Bioelectrical impedance analysis (BIA) provided additional insight into body composition. Fat mass accounted for approximately one quarter of body weight (25.4 ± 9.0%), while fat-free mass represented about three quarters (74.6 ± 9.9%). Total body water averaged 55.2 ± 9.9%, with intracellular water slightly exceeding extracellular water (53.6 ± 5.4% vs. 46.5 ± 5.4%). These values are consistent with a typical body composition profile in young adult women. Regarding menstrual cycle characteristics, 34 women reported a regular menstrual cycle and 15 reported irregular cycles. Comparisons between groups showed no statistically significant differences in age, height, weight, or BMI (all *p* > 0.05).

A similar pattern emerged for bioelectrical parameters. Resistance (R), reactance (Xc), phase angle (PA), fat-free mass (FFM), total body water (TBW), extracellular water (ECW), body cell mass (BCM), fat mass (FM), body cell mass index (BCMI), and fat-free mass index (FFMI) were comparable between women with regular and irregular cycles, with no statistically significant differences (all *p* > 0.05). Overall, menstrual cycle regularity was not associated with anthropometric or BIA-derived body composition variables. Anthropometric and bioelectrical impedance parameters by menstrual cycle regularity are presented in Table 1.

Lifestyle-related variables were also examined. No significant association was found between menstrual cycle regularity and physical activity levels (χ^2^ = 1.169, *p* > 0.05). Likewise, the number of daily meals and meal frequency (breakfast, lunch, snacks, dinner) were not associated with menstrual regularity (χ^2^ = 0.514, *p* > 0.05). Food intolerances were similarly unrelated to cycle regularity.

Dietary analysis revealed one statistically significant finding: meat consumption differed between groups, with lower consumption among women with irregular menstrual cycles compared to those with regular cycles (χ^2^ = 7.243, *p* = 0.007).

For all other food categories—including fish, eggs, dairy products, processed meats, vegetables, fruit, sweets, sugar-sweetened beverages, and coffee or tea—no statistically significant differences were observed (all *p* > 0.05). The associations between food consumption and menstrual cycle regularity are reported in Table 2.

Overall, these results indicate that, in this sample of young women, menstrual cycle regularity is not associated with differences in anthropometric characteristics, body composition, physical activity, or most dietary habits. The only dietary factor showing a significant association with menstrual regularity was meat consumption.

## 4. Discussion

The menstrual cycle reflects the complex hormonal regulation of the hypothalamic–pituitary–ovarian axis. Cyclical fluctuations in oestrogen and progesterone during the follicular and luteal phases regulate reproductive physiology through coordinated feedback mechanisms [23]. Lifestyle and nutritional factors can modulate these hormonal processes and may contribute to inter-individual variability in menstrual patterns [24]. Several biological mechanisms may explain the relationship between lifestyle, nutrition, and menstrual function [6,24]. Insulin resistance and metabolic dysregulation can directly affect ovarian steroidogenesis by stimulating androgen production and impairing follicular development [6,7]. Hyperinsulinemia may also reduce hepatic production of sex hormone-binding globulin, increasing circulating free androgens and contributing to ovulatory dysfunction [7]. In parallel, chronic low-grade inflammation and oxidative stress associated with unhealthy dietary patterns may influence hypothalamic signalling and gonadotropin secretion, further disrupting menstrual cyclicity [24,25]. Nutritional and metabolic factors may also affect endometrial physiology through modulation of estrogen metabolism, inflammatory pathways, and cellular proliferation, which may contribute to conditions such as abnormal uterine bleeding and endometrial hyperplasia [25,26]. Cycle-related conditions are increasingly viewed within a broader systemic context. Associations between gynecological disorders and migraine have been reported, suggesting shared neuroendocrine and inflammatory mechanisms in women’s health [27]. Menstrual irregularities are relatively common in adolescents and young women and are often functional rather than organic in origin. Although many menstrual alterations in young women are functional, structural uterine abnormalities may also contribute to reproductive disorders and infertility. Hysteroscopy represents the gold standard technique for the diagnosis and treatment of intrauterine abnormalities such as endometrial polyps, submucosal fibroids, uterine septa, and intrauterine adhesions, which may affect reproductive outcomes [28]. Diet, lifestyle, and body composition have been proposed as contributing factors to these functional alterations [25,29]. For this reason, investigating the relationship between nutritional status and cycle characteristics is clinically relevant from a preventive perspective. Previous research has suggested that dietary and lifestyle factors may influence menstrual physiology, and recent evidence indicates that adherence to dietary patterns and nutritional knowledge may also affect menstrual symptoms and distress [20]. Associations between dietary patterns, energy availability, and menstrual cycle regularity have been reported, supporting the biological plausibility of a link between diet and menstrual health [24,30,31]. In the present study, anthropometric parameters did not differ between women with regular and irregular menstrual cycles. Age, height, body weight, and BMI were comparable between groups, indicating that menstrual variability may occur even among women with normal body weight and apparently adequate general nutritional status. This finding highlights that irregular cycles can occur even in the absence of overt anthropometric alterations, suggesting that factors beyond body weight alone may be involved. Bioelectrical impedance parameters were also similar between groups. Although no statistically significant differences were observed, women with irregular cycles showed a tendency toward higher resistance and extracellular water, along with lower phase angle, intracellular water, and body cell mass. These patterns may reflect minor differences in hydration and cellular status [32,33]. Given that alterations in extracellular and intracellular water balance have been associated with inflammatory status and cellular integrity reported in earlier studies, even small variations may be physiologically relevant in the context of menstrual function. Since phase angle and body cell mass have been associated with nutritional and cellular health described in the literature, these observations are physiologically plausible, even if not statistically confirmed in this sample [34,35,36]. Regarding dietary habits, the most notable finding was the lower meat consumption reported by women with irregular cycles. While the cross-sectional design prevents causal interpretation, this result suggests that dietary quality and nutrient intake could play a role in menstrual function [26,37]. Meat represents a primary dietary source of highly bioavailable iron, zinc, and high-quality proteins, all of which are involved in ovarian function, steroidogenesis, and ovulatory regulation. Suboptimal intake of these nutrients may also affect hypothalamic–pituitary–ovarian axis activity. This hypothesis warrants further investigation in larger and longitudinal studies. No associations were found for other dietary variables, physical activity, or meal frequency. It is also possible that meat consumption acts as a proxy for overall dietary quality or micronutrient adequacy rather than representing an isolated dietary factor. This lack of association suggests that, in non-athlete populations with generally adequate lifestyle habits, menstrual regularity may not be strongly influenced by isolated lifestyle behaviors. Taken together, the findings indicate that menstrual regularity is likely influenced by multiple interacting factors rather than by single lifestyle components [25]. Overall, the findings indicate that menstrual patterns in young women may not be strongly related to basic anthropometric differences but could be associated with more subtle aspects of nutritional and body composition status. These observations support the concept that menstrual cycle regularity should be considered within a broader metabolic and reproductive health framework, in which lifestyle and nutritional factors may contribute to endocrine regulation, ovarian function, and endometrial physiology [6,25]. Early recognition and management of menstrual cycle regularity in adolescents and young women are considered important for long-term reproductive health [38]. Gynecological disorders have also been linked to systemic inflammatory states. For example, associations between endometriosis and inflammatory bowel disease have been reported, highlighting the complex interplay between reproductive and systemic health [39]. In this context, bioelectrical impedance analysis may serve as a complementary tool for evaluating body composition and hydration in young women, although its specific role in menstrual health assessment remains to be clarified. The relatively small sample size may have limited the statistical power to detect subtle associations. Furthermore, the cross-sectional design does not allow causal inference, and menstrual and dietary information was self-reported, which may be subject to recall bias. Hormonal measurements were not available, preventing endocrine correlations. In addition, information on potential confounding factors influencing menstrual regularity—such as hormonal contraceptive use, stress levels, sleep patterns, smoking, alcohol consumption, eating disorders, and specific dietary patterns (e.g., vegetarian or vegan diets or supplement use)—was not systematically collected. Moreover, no detailed clinical or biochemical screening was performed for endocrine conditions that may affect menstrual cycle regularity, such as thyroid dysfunction or hyperprolactinemia. Therefore, the presence of undiagnosed endocrine disorders cannot be excluded and may represent a potential source of confounding in the interpretation of menstrual cycle patterns. Overall, these factors should be considered when interpreting the results, and the present findings should be regarded as hypothesis-generating, providing preliminary insights into the relationship between nutrition and reproductive health in this population.

## 5. Conclusions

This study evaluated the relationship between nutritional status, body composition, and menstrual cycle characteristics in young women. Menstrual regularity was not linked to major anthropometric differences, indicating that cycle variability can occur even in women with normal body weight. Although bioelectrical impedance parameters were similar between groups, trends in hydration and cellular indicators suggest that subtle body composition differences may be relevant to menstrual health. Lower meat consumption was associated with menstrual irregularity, pointing to a possible role of dietary quality in reproductive function. While causality cannot be established, this finding aligns with emerging evidence on the nutrition–menstrual health relationship. Overall, menstrual health should be considered within a broader nutritional and lifestyle framework. Longitudinal studies are needed to better define these associations.

## Figures and Tables

**Table 1:** Anthropometric and bioelectrical impedance parameters according to menstrual cycle regularity. Values are expressed as mean ± SD. No significant differences were observed between groups (all *p* > 0.05).

Variable	Menstrual Cycle	*N*	Mean	SD	Min	Max	*p* (ANOVA)
Age (years)	Irregular	15	21.6	1.5	19	24	0.811
	Regular	34	21.7	1.9	19	27	
Height (cm)	Irregular	15	166.0	9.1	155	183	0.470
	Regular	34	164.4	6.4	153	177	
Weight (kg)	Irregular	15	61.2	13.5	46	100	0.899
	Regular	34	60.7	12.1	43	95	
BMI (kg/m^2^)	Irregular	15	22.0	3.0	17.1	30.5	0.714
	Regular	34	22.5	4.7	17.7	40.6	
Resistance (Ω)	Irregular	15	623.7	96.6	447	812	0.592
	Regular	34	611.3	62.3	490	800	
Reactance (Ω)	Irregular	15	61.1	9.7	50	77	0.744
	Regular	34	62.0	8.0	46	80	
Phase angle (°)	Irregular	15	5.6	0.6	4.3	6.6	0.320
	Regular	34	5.8	0.6	4.1	7.3	
Fat-free mass (kg)	Irregular	15	45.0	8.9	36.8	72.0	0.807
	Regular	34	44.5	5.6	37.2	67.8	
Total body water (L)	Irregular	15	32.8	6.7	27.0	52.9	0.854
	Regular	34	33.3	8.5	27.0	78.4	
Extracellular water (L)	Irregular	15	15.6	2.6	12.2	22.7	0.262
	Regular	34	14.9	1.6	12.2	18.1	
Body cell mass (kg)	Irregular	15	23.4	5.7	16.1	40.8	0.686
	Regular	34	24.2	7.5	17.4	63.8	
Fat mass (kg)	Irregular	15	16.2	6.3	6.2	28.0	0.994
	Regular	34	16.2	10.0	2.2	49.4	
BCM index (kg/m^2^)	Irregular	15	8.4	1.4	6.6	12.5	0.443
	Regular	34	9.0	2.6	6.5	22.6	
Fat-free mass index (kg/m^2^)	Irregular	15	16.2	2.0	13.4	22.0	0.680
	Regular	34	16.5	1.8	13.9	24.0	

**Table 2:** Association between food consumption and menstrual cycle regularity. Values are reported as n/N (%) within each menstrual cycle group. Group differences were assessed using the chi-square test or Fisher’s exact test, as appropriate.

Food	% Not Consumed (Regular)	% Consumed (Regular)	% Not Consumed (Irregular)	% Consumed (Irregular)	χ^2^ (*p*-Value)
Meat	0% (0/34)	100% (34/34)	20.0% (3/15)	80.0% (12/15)	7.243 (0.007)
Fish	8.8% (3/34)	91.2% (31/34)	6.7% (1/15)	93.3% (14/15)	0.065 (0.799)
Processed meats	29.4% (10/34)	70.6% (24/34)	20.0% (3/15)	80.0% (12/15)	0.473 (0.492)
Eggs	11.8% (4/34)	88.2% (30/34)	6.7% (1/15)	93.3% (14/15)	0.295 (0.587)
Dairy products	5.9% (2/34)	94.1% (32/34)	20.0% (3/15)	80.0% (12/15)	2.264 (0.132)

## Data Availability

The data that support the findings of this study are available from the corresponding author, E.P., upon reasonable request.
